# Mapping of Current Obstacles for Rationalizing Use of Medicines (CORUM) in Europe: Current Situation and Potential Solutions

**DOI:** 10.3389/fphar.2020.00144

**Published:** 2020-03-03

**Authors:** Mohamed Gad, Ahmed Salem, Wija Oortwijn, Ruaraidh Hill, Brian Godman

**Affiliations:** ^1^Global Health and Development Group, Imperial College London, London, United Kingdom; ^2^Real World Evidence Solutions, IQVIA, Zaventem, Belgium; ^3^Department for Health Evidence, Radboud University Medical Center, Nijmegen, Netherlands; ^4^Liverpool Reviews and Implementation Group, University of Liverpool, Liverpool, United Kingdom; ^5^Department of Laboratory Medicine, Division of Clinical Pharmacology, Karolinska Institutet, Karolinska University Hospital Huddinge, Stockholm, Sweden; ^6^Strathclyde Institute of Pharmacy and Biomedical Sciences, University of Strathclyde, Glasgow, United Kingdom; ^7^Health Economics Centre, University of Liverpool Management School, Liverpool, United Kingdom; ^8^Department of Public Health Pharmacy and Management, School of Pharmacy, Sefako Makgatho Health Sciences University, Garankuwa, South Africa

**Keywords:** rational use of medicines, Europe, health authorities, initiatives, health technology assessment

## Abstract

**Introduction:**

There are increasing concerns regarding the inappropriate use of medicines with expenditure continuing to grow driven by increasing sales in oncology and orphan diseases, enhanced by their emotive nature. As a result, even high income countries are struggling to fund new premium priced medicines. These concerns have resulted in initiatives to better manage the entry of new medicines and enhance the rational use of medicines (RUM). However, there is a need to ascertain the current situation. We sought to address this by developing the Current Obstacles for Rationalizing Use of Medicines in Europe (CORUM) mapping tool to qualitatively investigate the current situation and provide analysis of current views on RUM and interventions among key European payers and their advisers. The findings will be used to provide future guidance.

**Methodology:**

Descriptive study exploring and identifying perceived gaps to achieving optimal RUM. The CORUM tool was based on the WHO 12 key interventions to promote RUM.

**Results:**

62 participants took part with most respondents believing their country could improve RUM capacity. This included educational initiatives on the use of clinical guidelines (90%) and the inclusion of problem-based pharmacotherapy in undergraduate curricula and for Continued Professional Development. Key challenges included a lack of regular updates of guidelines, exacerbated by limited funding and a lack of follow-up to monitor adherence to agreed guidelines. RUM could also be enhanced by the development of regional formularies as well as implementing Drug and Therapeutic Committees where these are currently limited. There also needs to be greater co-ordination between RUM and Health Technology Assessment activities, with countries learning from each other.

**Conclusion:**

There is an urgent need to improve RUM through improved educational and other activities among European countries, with countries learning from each other. This will involve addressing current challenges and we will be following this up.

## Introduction

The World Health Organization (WHO) estimates that more than half of all medicines are prescribed, dispensed or sold inappropriately, and that half of all patients fail to take them correctly (World Health Organization, 2012; Ofori-Asenso and Agyeman, 2016). This results in the waste of healthcare resources as well as leads to health hazards. The situation is even more critical in lower- and middle-income countries (LMICs) where expenditure on medicines can be up to 70% of total healthcare expenditure, much of which is out-of-pocket (Cameron et al., 2009; Ofori-Asenso and Agyeman, 2016). Expenditure on medicines has increased since 2015 beyond US$800 billion (€720 billion) among countries of The Organisation for Economic Co-operation (OECD) (European Central Bank, 2015; OECD, 2017). This is driven by increased expenditure on biological medicines, including those for cancer, as well as new medicines for patients with hepatitis C (OECD, 2017). Total retail pharmaceutical expenditure in Europe was more than €210 billion in 2016 (adjusting for purchasing power parities), an increase of 5% since 2010 (OECD/EU, 2018). The increase was limited by strategies across Europe to enhance the prescribing of low-cost generics and biosimilars versus originators and patented products where care is not compromised (Godman et al., 2014a; Moon et al., 2014; Moorkens et al., 2017; Vogler and Schneider, 2017; OECD/EU, 2018; Simoens et al., 2018; Godman et al., 2019a).

Expenditure on cancer medicines is a concern, with global expenditure in 2017 at US$133 billion (~€ 117 bn) up from US$96 billion in 2009, and estimated to reach US$200 billion (~ € 176 bn) by 2022 with an average growth rate of 10 to 13% per year during the coming years (IQVIA Institute for Human Data Science, 2018). This increase in expenditure will be driven by anticipated increases in the prices of new cancer medicines and increased incidence rates with an estimated 21.4 million new cancer cases globally per year by 2030 up from 18.1 million in 2018 (Chalkidou et al., 2014; Kelly and Smith, 2014; Howard et al., 2015; Godman et al., 2017; Bray et al., 2018). In addition, over 500 companies are actively pursuing new cancer medicines across multiple indications (IMS Institute for Healthcare Informatics, 2016; Godman et al., 2018), intensifying expectations for the rising costs for oncology medicines (Durkee et al., 2016; Haycox, 2016; Godman et al., 2017; Dolgin, 2018). There are similar concerns and issues with new medicines for patients with orphan diseases (Simoens et al., 2013; Godman et al., 2018; Luzzatto et al., 2018) where we are likely to see global spending on orphan medicines reaching US$178 billion (~€ 160 bn) per year by 2020 (Brau and Tzeng, 2018). This will again be enhanced by the number of medicines for orphan diseases currently available and in development, and their envisaged prices (Godman et al., 2016; Godman et al., 2018; Luzzatto et al., 2018).

Overall, the costs of new medicines are a growing concern across countries driven by changing demographics, rising patient expectations, single disease treatment guidelines, and the continued launch of new premium priced medicines (WHO, 2015; Godman et al., 2014b; Godman et al., 2018). This is set to continue exacerbated by the number of new medicines in development as well as the emotive nature of disease areas such as cancer and orphan diseases (Simoens et al., 2013; WHO, 2015; Haycox, 2016; Cohen, 2017; Goldstein et al., 2017; Godman et al., 2018; Luzzatto et al., 2018). We are also seeing high income countries increasingly struggle to fund new medicines, and this will continue unless actively addressed (Malmstrom et al., 2013; Ghinea et al., 2015; Godman et al., 2018; Kwon et al., 2018). These challenges have led to the development of new models to better manage the entry of new medicines as well as initiatives to enhance the rational use of medicines (RUM). These new models include encouraging the use of biosimilars and multiple sourced products where pertinent to conserve resources alongside instigating active disinvestment processes (Godman et al., 2014a; WHO, 2015; Parkinson et al., 2015; Guerra-Junior et al., 2017; Moorkens et al., 2017; Godman et al., 2018) as well as a growing use of managed entry agreements (MEAs). However, there are concerns with MEAs including the extent and usefulness of any clinical data collected, as well as the ability to actively monitor the role and value of new medicines in routine clinical care to provide future guidance (Ferrario and Kanavos, 2013; WHO, 2015; Garattini and Curto, 2016; Carlson et al., 2017; Ferrario et al., 2017; Garcia-Doval et al., 2017; Guerra-Junior et al., 2017; Mercer et al., 2017; Eriksson et al., 2018; Frisk et al., 2018; Alvarez-Madrazo et al., 2019; Antonanzas et al., 2019; Mueller et al., 2019).

The WHO defines RUM as “Patients receive medications appropriate to their clinical needs, in doses that meet their own individual requirements, for an adequate period of time, and at the lowest cost to them and their community” (World Health Organization, 2002; World Health Organization, 2007). According to the WHO, a sound rational drug use program in any country involves interventions on three levels. The first is on the level of healthcare authorities that includes setting strategies for monitoring the use of medicines, second is on the level of healthcare service providers, which for example includes enhancing the use of agreed clinical guidelines, and the third is on the level of medicines used by consumers, which includes raising awareness on key aspects of RUM (World Health Organization, 2012). Whilst these strategies are operational in a number of countries, they are rarely subjected to a thorough assessment of their actual impact in practice such as encouraging the preferential use of multiple sourced medicines where pertinent without compromising care or encouraging caution for new medicines where there are concerns with patient safety (Godman et al., 2010a; Wettermark et al., 2010; Forslund et al., 2011; Kaplan et al., 2012; Moon et al., 2014; Godman et al., 2014b; Troncoso and Diogene, 2014; Frisk et al., 2018; Jacobs et al., 2019). Especially, there seems to be a gap regarding stakeholder engagement to assess the implementation of these strategies, or in gathering information about the key challenges of implementation and ways of circumventing these challenges, with a focus on variable contexts and different healthcare ecosystems.

We sought to address this gap by developing the Current Obstacles for Rationalizing Use of Medicines in Europe (CORUM) mapping tool. The objective of the CORUM tool was to qualitatively investigate the current situation and to provide an in-depth analysis of current views on RUM interventions among key payers and their advisers among European countries in addition to the challenges encountered when attempting to implement these interventions. The findings will be used to develop strategies to enhance RUM in Europe given increasing budgetary and other concerns.

## Methodology

### General

This is a descriptive study exploring and identifying perceived gaps to achieving optimal RUM in Europe. The CORUM tool (**Supplementary Appendix 1**) was developed by the authors based on the WHO 12 key interventions to promote RUM (**Table 1**) (World Health Organization, 2012). For each key intervention, we inquired regarding the extent to which respondents agreed to the intervention being a key intervention to promote RUM, checked for current obstacles for this intervention to be realized based on respondents’ experience in his/her own country, and asked for respondents’ views concerning possible ways to circumvent these obstacles to enhance the rational use of medicine within their own country (**Supplementary Appendix 1**). The study also embeds considerations from common elements including cooperation between national organizations that are traditionally evident in Health Technology Assessment (HTA) frameworks widely established within member states of the European Union (EU) (EUnetHTA, 2018; EUnetHTA Mission, vision and values, 2019; Vella Bonanno et al., 2019). HTA is the multidisciplinary process to determine the value of a health technology. The dimensions of value for a health technology may be assessed by examining intended and unintended consequences of health technologies, including the clinical, economical, ethical, social, organizational, and environmental aspects, as well as wider implications for the patient, relatives, caregivers, and the population. The overall value may vary depending on the perspective taken, the stakeholders involved, and the decision context.

**Table 1:** WHO’s 12 Key interventions to promote rational use of medicine.

1. Establishment of a multidisciplinary national body to coordinate policies on medicine use
2. Use of clinical guidelines
3. Development and use of national essential medicines lists
4. Establishment of drug and therapeutics committees in districts and hospitals
5. Inclusion of problem-based pharmacotherapy training in undergraduate curricula
6. Continuing in-service medical education as a licensure requirement
7. Supervision, audit, and feedback
8. Use of independent information on medicines
9. Public education about medicines
10. Avoidance of perverse financial incentives
11. Use of appropriate and enforced regulation
12. Sufficient government expenditure to ensure availability of medicines and staff.

We believe this combination of sources was an appropriate starting point to develop a cross-sectional survey (see *CORUM Mapping Survey*) that could be distributed across key stakeholders in Europe to gather their perception of the proposed RUM interventions.

### Participants

The target audience of the tool could be categorized into two main groups. The first were the policy makers (users of evidence) who are typically the decision makers responsible for legislating and implementing frameworks for adopting and monitoring the use of medicines within their own region or country. The second group were “technocrats” (suppliers of evidence), who are often the producers of assessments of medicines or those giving recommendations that directly or indirectly influence decisions regarding RUM based on evidence-based medicine and/or HTA (Ward et al., 2009).

Sampling for the participants to be surveyed was conducted based on a purposive non-random sampling technique, which is typically used for exploratory work such as this study. The participants who were approached for this initial survey were stakeholders across various realms of the healthcare community spread across different geographical locations within Europe. The participants were primarily members of Piperska Group, a non-governmental organization which the authors are also members.

The Piperska Group is a pan-European network of payers (health insurers), advisers, and academics that researches and analyzes key health policies and their impact with the aim of enhancing the health of populations and individual patients in a sustainable way (Garattini et al., 2008). Activities include developing new models to improve the managed entry of new medicines, developing quality indicators for new medicines, assessing key issues such as MEAs, discussing minimum effectiveness levels for new cancer medicines, appraising key issues surrounding personalized medicine and adaptive pathways as well as potential ways to enhance the prescribing of low cost generics and biosimilars to release resources to fund increased medicine volumes with aging populations and new valued medicines within agreed budgets (Godman et al., 2013a; Malmstrom et al., 2013; Godman et al., 2014b; Godman et al., 2014c; Moon et al., 2014; Campbell et al., 2015; Godman et al., 2015a; de Bruijn et al., 2016; Ermisch et al., 2016; Wild et al., 2016; Ferrario et al., 2017; Moorkens et al., 2017; Vella Bonanno et al., 2017; Godman et al., 2018; Godman et al., 2019b; Vella Bonanno et al., 2019). In addition, organizing courses to improve the managed entry of new medicines and prescribing at the interface (Godman et al., 2012; Bjorkhem-Bergman et al., 2013; Matusewicz et al., 2015). The various activities and outputs have been achieved through cooperation and the exchange of ideas among a wide base of multi-stakeholder groups in Europe. As a result, this group helps provide European thought leadership specifically for constructing policies around enhancing RUM and related therapies.

Consequently, the Piperska Group was deemed an appropriate target audience to drive this exploratory mapping study. Furthermore, we also incorporated a snowball sampling technique where existing study participants invited future participants to take part in this survey. Initially, prospective participants were approached through e-mail, inviting them to participate in the survey with the potential of forwarding the survey to known colleagues.

### Tool: CORUM Mapping Survey

#### Development of the Survey

The CORUM Mapping Tool is a survey of 9 structured questions where each question pertains to one of the WHO key interventions. Although the WHO proposes 12 key interventions for optimal RUM, we adapted the questions to combine one or more interventions to avoid a lengthy questionnaire. The authors established face-validity of the survey by allowing experts in the field including health authority personnel to read through the survey and share their feedback. The survey was subsequently reviewed by a psychometrician to check for common questionnaire errors. We adjusted the survey based on the received feedback and subsequently ran a pilot test on a subset of the prospective participants to enhance the robustness of the survey. The final survey is available as a supplement.

A brief introduction about the survey, its objectives, and the instructions were contained in the cover page. Each question in the survey first presented one of the key interventions and subsequently asked the participants to select a score on a 1–5 scale that best represented how strongly they agreed (score 5) or disagreed (score 1) that the particular intervention is key for RUM. A 5-point scale is standard tool for research studies such as this one (Desser et al., 2010; Shrank et al., 2011; Deutsch et al., 2015; Garcia et al., 2019). Thereafter, the participants rated their country’s performance with regards to the presented key intervention. Following this, the participants selected whether or not they believed there are current challenges to realizing the presented intervention in addition to clarifying what these challenges are. The final sub-question asked the participants to propose possible solutions to overcome perceived challenges to implementing the presented intervention.

We used the online website checkmarket.com to build the questionnaire layout and subsequently deliver it to the participants. Checkmarket offers a user-friendly platform to creating, administering, and analyzing online surveys. The survey ran for 3 months between March and June 2017 although we allowed one week prior to the “active period” where we sent the invitation e-mails to participants inviting them to take part. Weekly reminders were sent to participants requesting them to complete the survey or clarify any questions if they had any. All communications with participants were conducted through e-mail.

#### Analysis

The responses were analyzed to present a frequency distribution of the answers. We typically consolidated the high-end and low-end scores in the 1–5 point scale, e.g. strongly disagree and disagree, for ease of analysis.

The same approach was followed for questions with binary answers (i.e. yes or no) and multi-select questions. Finally, the open-answers, where the participants stated possible ways to address the perceived challenges of achieving optimal rational use of medicines, were coded and analyzed according to the so-called 4 E approach (Education, Economics, Engineering, and Enforcement) first discussed by Wettermark et al. (2009a) and used extensively since then to analyze the influence of typically multiple health policy interventions to improve the quality and/or efficiency of prescribing (Wettermark et al., 2009a; Godman et al., 2010a; Godman et al., 2010b; Voncina et al., 2011; Woerkom et al., 2012; Fraeyman et al., 2013; Godman et al., 2013b; Moon et al., 2014; Leporowski et al., 2018; McCabe et al., 2019).

The 4 E’s consolidate the multiple strategies that can be applied to rationalizing medicine use. Education refers to a wide range of educational activities ranging from simple distribution of printed material to more advanced strategies such as academic detailing and educational visits by trained facilitators. Academic detailing, sometimes called educational outreach visits, typically includes a visit of a trained health professional such as a physician or pharmacist to a general practitioner in their own setting on a one-on-one basis to provide information to help change behavior (Grimshaw et al., 2004; O’Brien et al., 2007; Jin et al., 2012; Chhina et al., 2013; Costa et al., 2016). Goals can include active dissemination and discussion on agreed national or regional treatment guidelines and improving the management of targeted health conditions (Chhina et al., 2013; Godman et al., 2014d; Costa et al., 2016). Typically academic detailing is undertaken in one or more visits and often combined with other strategies to enhance its effectiveness including audits and other feedback approaches (O’Brien et al., 2007; Gustafsson et al., 2011; Costa et al., 2016).

Engineering captures strategies that are more organizational or managerial interventions such as monitoring of prescribing against agreed targets such as the quality and outcome framework in the United Kingdom or percentage targets for prescribing in Scotland or Sweden (Godman et al., 2009; McGinn et al., 2010; Campbell et al., 2011; Godman et al., 2014a). Economics strategies typically include financial incentives or disincentives to all key stakeholder groups including physicians, pharmacists, and patients, i.e. financial incentives for achieving agreed prescribing targets and writing annual reports on potential ways to improve future prescribing (Godman et al., 2009; Wettermark et al., 2009b; McGinn et al., 2010; Martin et al., 2014). Finally, Enforcement strategies are those that include regulations by law such as mandatory generic substitution or mandatory prescribing restrictions (Godman et al., 2009; Wettermark et al., 2009a; Wettermark et al., 2010; Godman et al., 2014d).

We did not seek ethical approval as this study did not involve patients. This is in line with previous studies undertaken by the co-authors in related areas including analysis of policies to enhance the rationale use of medicines as well as seeking a greater role for biosimilars and generics involving health authority personnel and their advisers (Godman et al., 2010a; Godman et al., 2010b; Godman et al., 2014c; Moon et al., 2014; Godman et al., 2015b; Ferrario et al., 2017; Moorkens et al., 2017; Godman et al., 2019b) as well as in line with local legislation and institutional guidelines. Nevertheless, sufficient information was provided to participants about the objectives of this study in an invitation email and within the survey instrument (see **Supplementary Appendix 1**), with consent sought to gather responses and analyse the results for research purposes.

## Results

### Response Rate

The link to the survey was eventually emailed to 235 potential respondents. Overall, 62 professionals completed the questionnaire giving a response rate of 26% which is in line with the average response rates for e-mail surveys (Fincham, 2008).

**Table 2** below shows the breakdown of respondents (n = 62) by professional category and European region. The majority of the participants were from national health organization bodies (n = 20) followed by academics (n = 19), while the highest portion of respondents were from Eastern Europe (n = 37). There was also a high percentage of Piperska member respondents in the health insurance and national health organization group (56%), with the least number among healthcare professionals (17%).

**Table 2:** Survey Respondents.

Participant Category	CE	EE	NE	SE	TOTAL, n (%)
Health Insurer	1	2	1	0	4 (7)
Industry	0	7	0	0	7 (11)
National Health Organization	2	10	5	3	20 (32)
Health Care Professional	2	5	3	2	12 (19)
University/Academic	2	13	3	1	19 (31)
TOTAL, n (%)	7 (11)	37 (60)	12 (19)	6 (10)	62 (100)

CE, Central Europe; EE, Eastern Europe; NE, Northern Europe; SE, Southern Europe.

### Capacity for Rational Use of Medicine

**Figure 1:**
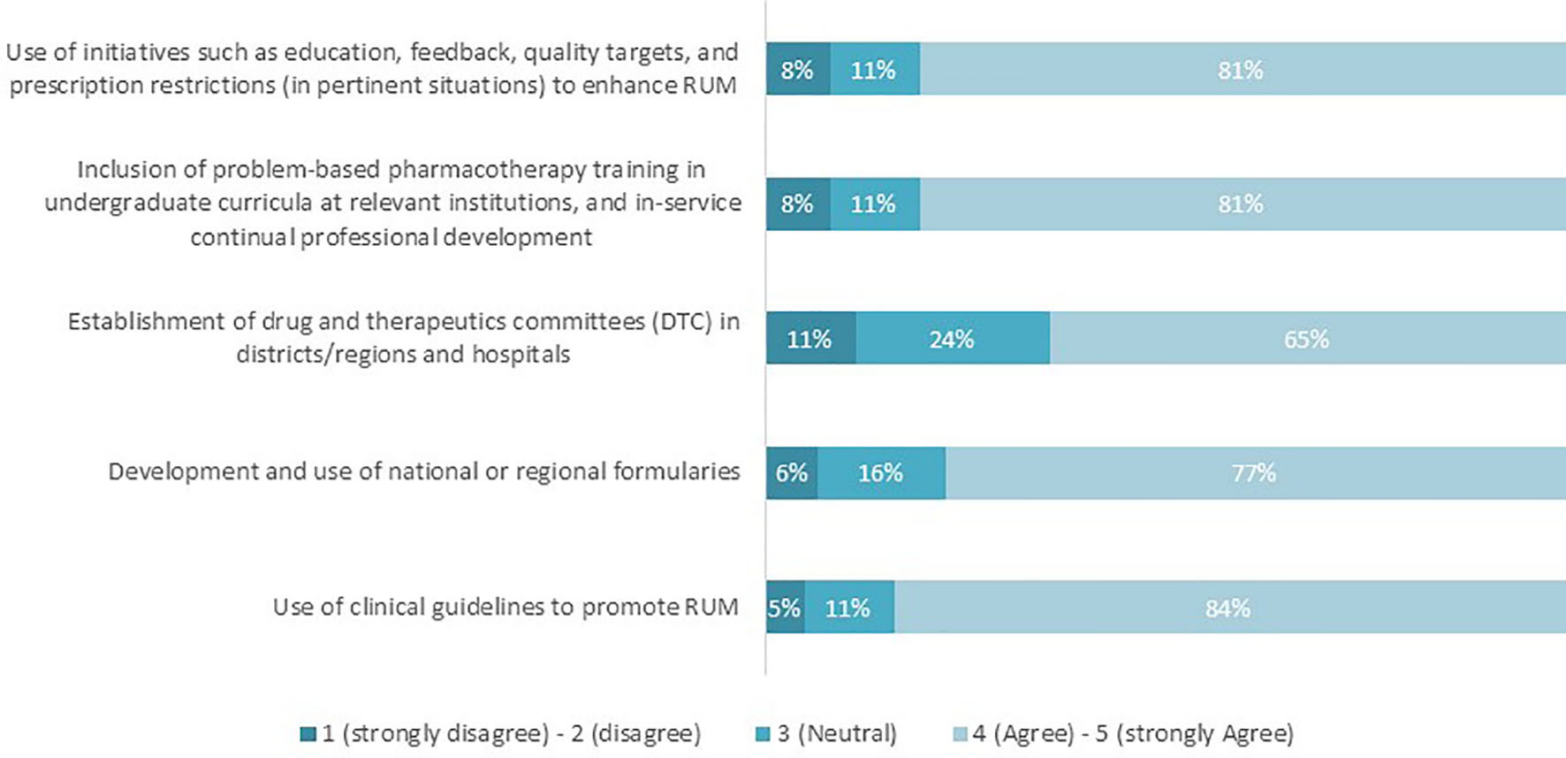
Proportion of respondents who agree/disagree that the interventions listed promote RUM (n = 62).

The majority of the respondents agreed that the presented five interventions are perceived as key contributors for RUM (see **Figure 1**). The percentage of agreement ranged from 65% (drug therapeutic committees, DTCs) to 84% (clinical guidelines).

**Figure 2:**
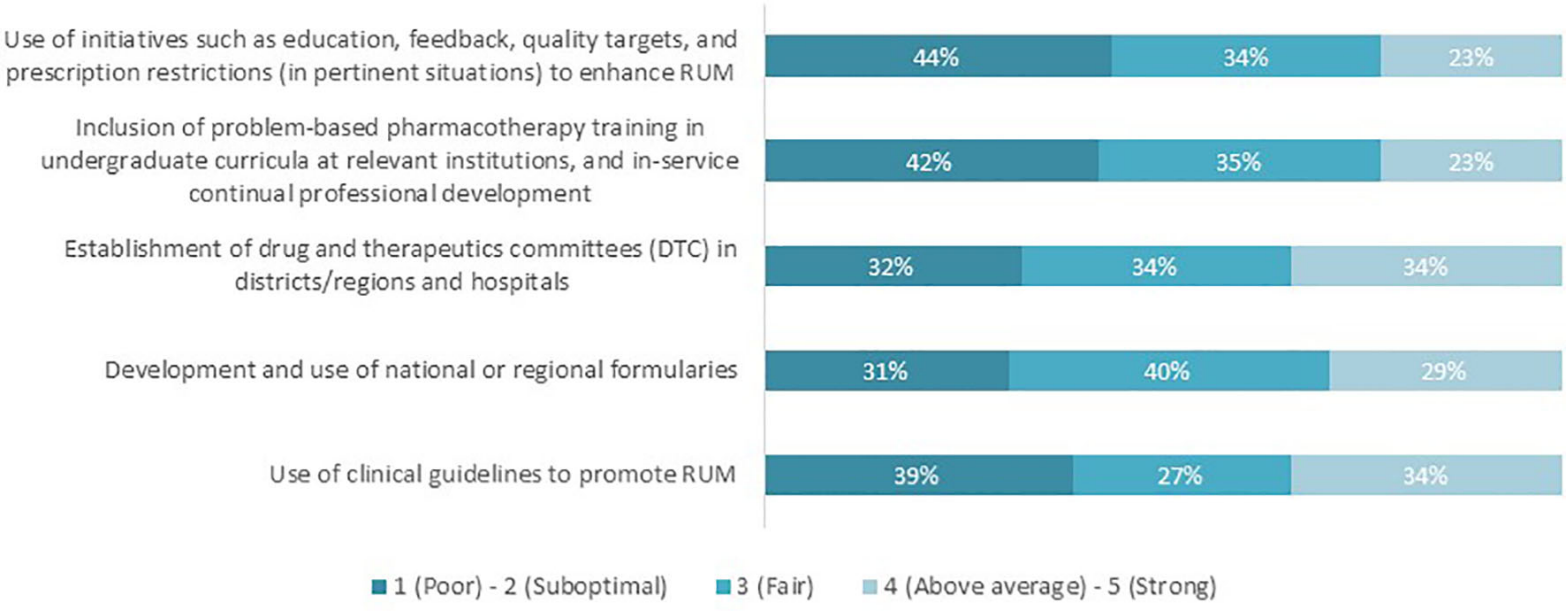
Countries’ performance in achieving the distinct key interventions to promote RUM, as perceived by the respondents (n = 62).

Respondents subsequently scored the performance of their countries in achieving the relevant key RUM interventions on a scale of 1–5, where 1 refers to “Poor performance” and 5 being “Strong performance”. The percentage of respondents who believed that their country performed “above average” or “strongly” ranged between 23 and 34% across all interventions (**Figure 2**). The remaining respondents (66–77%) believed that their country’s performance with achieving RUM capacity was currently poor, suboptimal, or fair.

**Figure 3:**
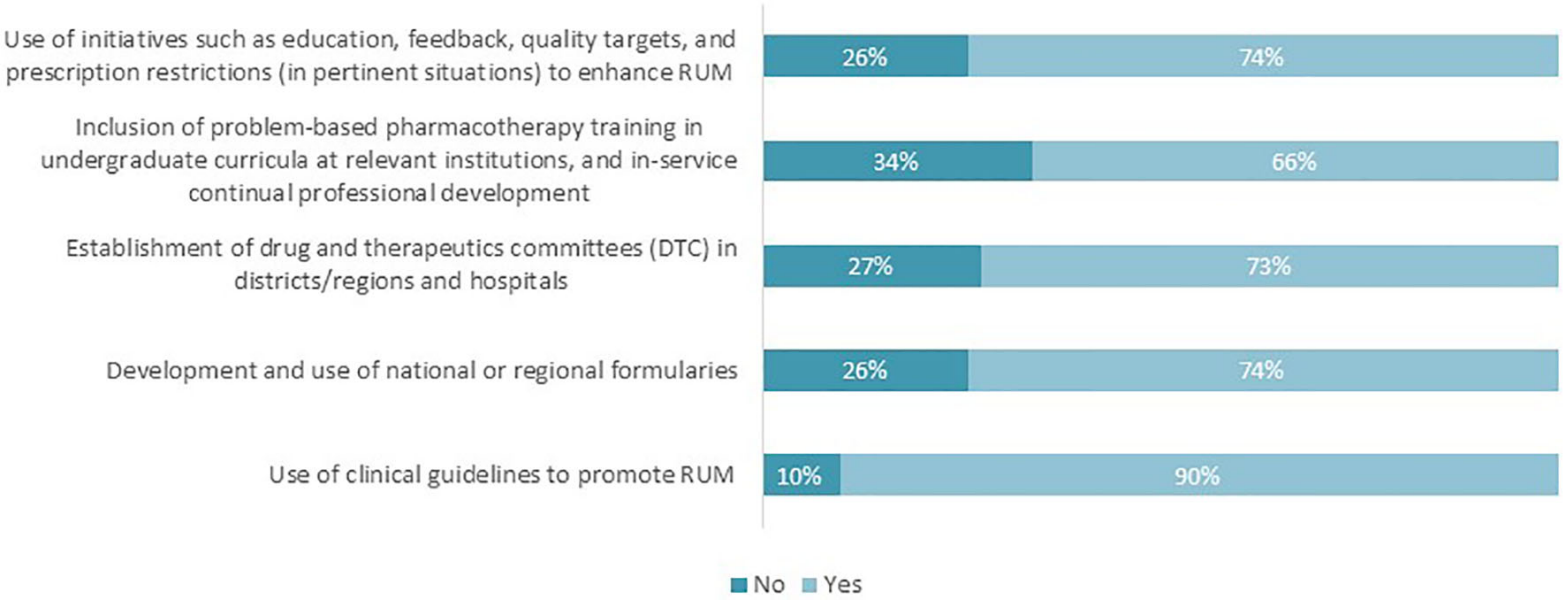
Proportion of respondents perceiving that there are challenges to realizing the key interventions to promote RUM (n = 62).

The majority of the respondents highlighted that there are current challenges to instigating appropriate interventions to enhance RUM (**Figure 3**). The percentages ranged from 66% regarding the inclusion of problem-based pharmacotherapy training to 90% with regard to the use of clinical guidelines.

**Figure 4:**
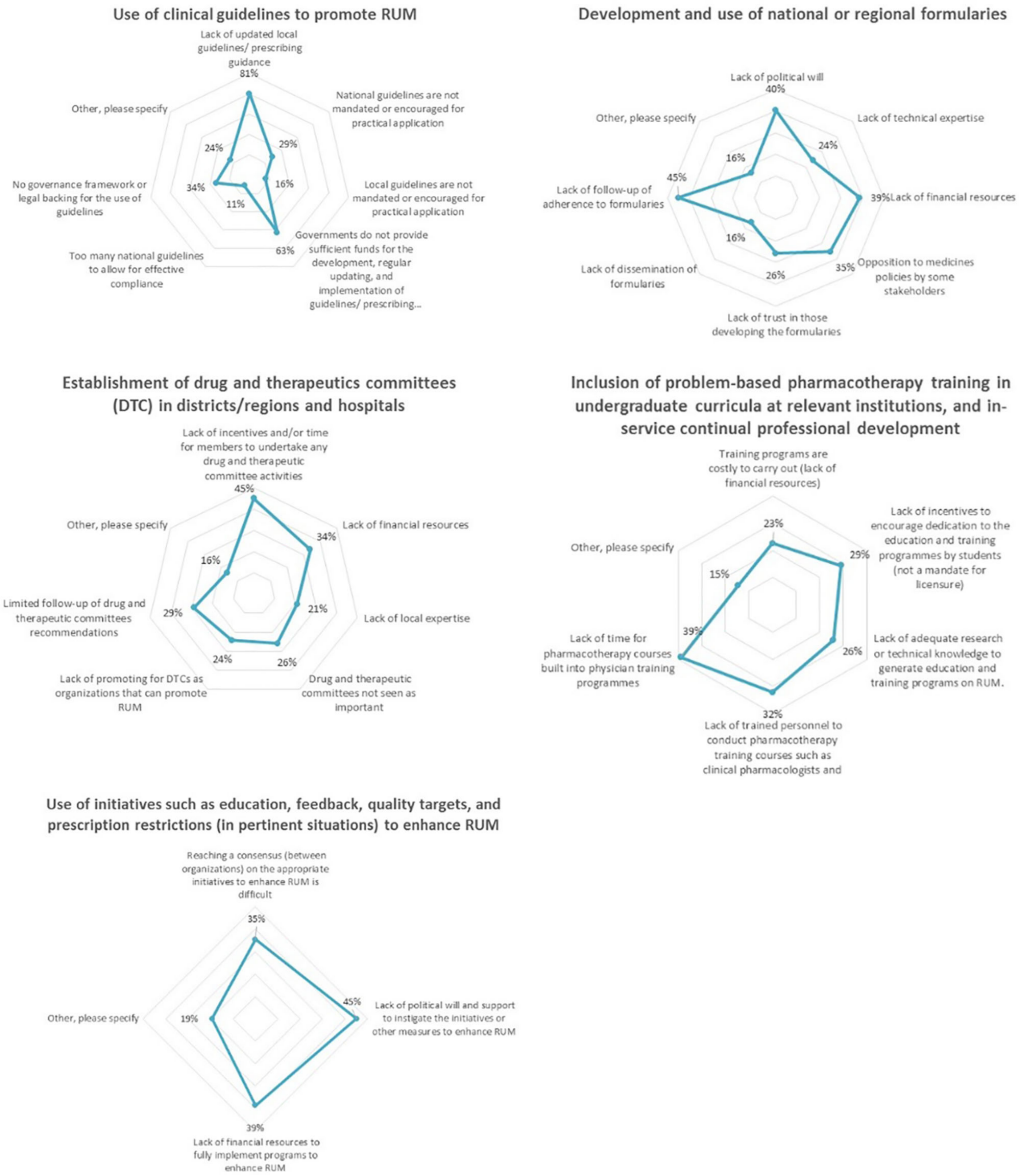
Current challenges to the implementation of key interventions aimed to promote RUM (n = 62).

The two most selected challenges hindering the use of clinical guidelines were “lack of updated guidelines or prescribed guidance” and lack of “governmental funding” to maintaining these guidelines (**Figure 4**). “Lack of follow-up of adherence to formularies” was regarded as the greatest challenge to the development and use of national/regional formularies. Furthermore, 45% of the respondents selected “lack of incentives and/or time” as a limitation to establishing DTCs in districts, regions, and hospitals. Of the respondents, 39% cited “lack of time” as a challenge to including problem-based pharmacotherapy training in undergraduate courses, while 45% of the respondents believed that the lack of government support hinders initiatives that support RUM such as education, feedback, and prescription restrictions.

**Figure 5:**
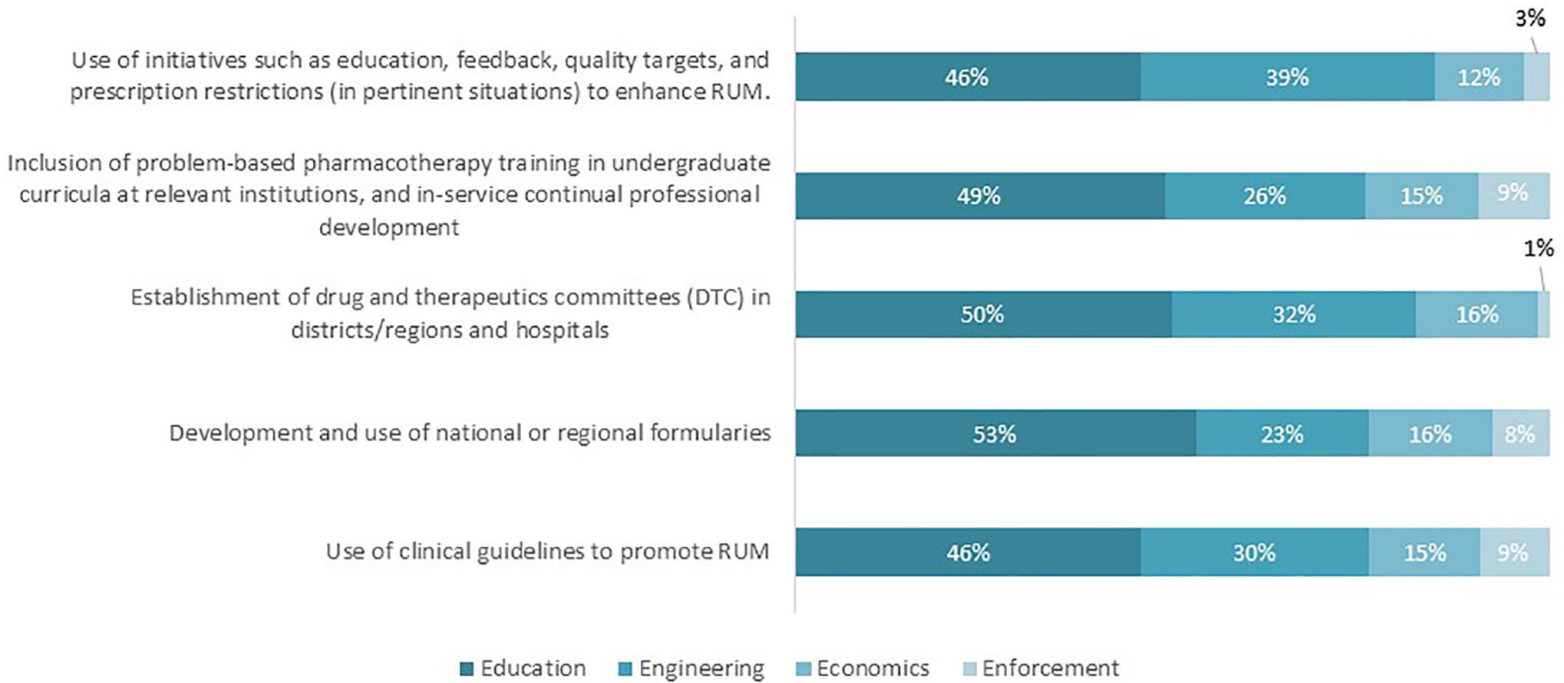
Possible solutions to address identified challenges regarding the key interventions to promote RUM (n = 62).

**Figure 5** shows the possible solutions proposed by the respondents to overcome current challenges to promoting RUM. “Education” was the solution most proposed by the respondents (46–53%). Proposed activities included creating RUM guidelines, implementing electronic aids to support prescription writing and knowledge sharing through congresses. Initiatives that are categorized as “Engineering” approaches were the second most proposed strategies to address potential challenges, which include stronger involvement of public health authorities and national payers in promoting RUM strategies. Finally, strategies pertaining to “Economics” and “Enforcement” solutions were the least proposed by participants. Examples of these strategies are increased governmental funding for the implementation of guidelines including incentives to physicians (Economics) and payer contractual agreements with prescribers that guidance must be adhered to (Enforcement).

### Linkage Between HTA and RUM

We asked respondents what kind of body is responsible for coordinating RUM initiatives and/or policies in their countries. Fifty-six percent (n = 35) of total respondents stated that their country has a body that coordinates RUM initiatives and/or policies. Among these, the majority of bodies were governmental national units (HTA entity) within the ministry of health (MoH) (23%, **Table 3**). However, 23% of respondents mentioned that the HTA entity in their respective country (if this exists) is not involved in coordinating RUM activities. The other selected types of RUM-coordinating entities varied between being independent governmental agencies (13%), regulatory governmental authority (8%), or a payer agency (6%). Furthermore, 85% (n = 53) of respondents were aware of WHO Europe as a pan-European stakeholder which advocates RUM (**Table 4**), followed by EuNetHTA (68%, n = 42) and Piperska Group (65%, n = 40).

**Table 3:** Types of HTA entities that are involved with RUM policy initiatives and/or coordination as per respondents’ answers (n = 62).

Type of HTA entity	Proportion of respondents, % (n)
No HTA entity involved with RUM policy coordination	23% (14)
Government national unit (within MoH)	23% (14)
Government agency (independent)	13% (8)
Regulatory authority national government	8% (5)
Funding agency (i.e. payer)	6% (4)
Professional society (national)	5% (3)
Academia	5% (3)
More than one entity	5% (3)
Network of HTA agencies (more than one country)	3% (2)
Healthcare provider under MoH (e.g. public hospital)	3% (2)
Regional drug agency	2% (1)
Public insurance agency	2% (1)
Professional society—international	2% (1)
Network of HTA agencies (within one country)	2% (1)

HTA, health technology assessment; RUM, rational use of medicine; MoH, Ministry of Health.

**Table 4:** Proportion of respondents who are aware of the different Pan-European groups advocating RUM (n = 62).

Pan-European Group	Proportion of respondents aware, % (n)
WHO Europe	85% (53)
EuNetHTA	68% (42)
Piperska Group	65% (40)
MEDEV	42% (26)
HTAi	40% (25)
EuroDURG	37% (23)
PPRI	37% (23)
EACPT	18% (11)
Others	13% (8)

WHO, World Health Organization; EuNetHTA, European network for health technology assessment; MEDEV, The Medicine Evaluation Committee; HTAi, Health Technology Assessment International; EuroDURG, European Drug Utilisation Research Group; PPRI, Pharmaceutical pricing and reimbursement information initiative; EACPT, European Association for Clinical Pharmacology and Therapeutics.

**Figure 6:**
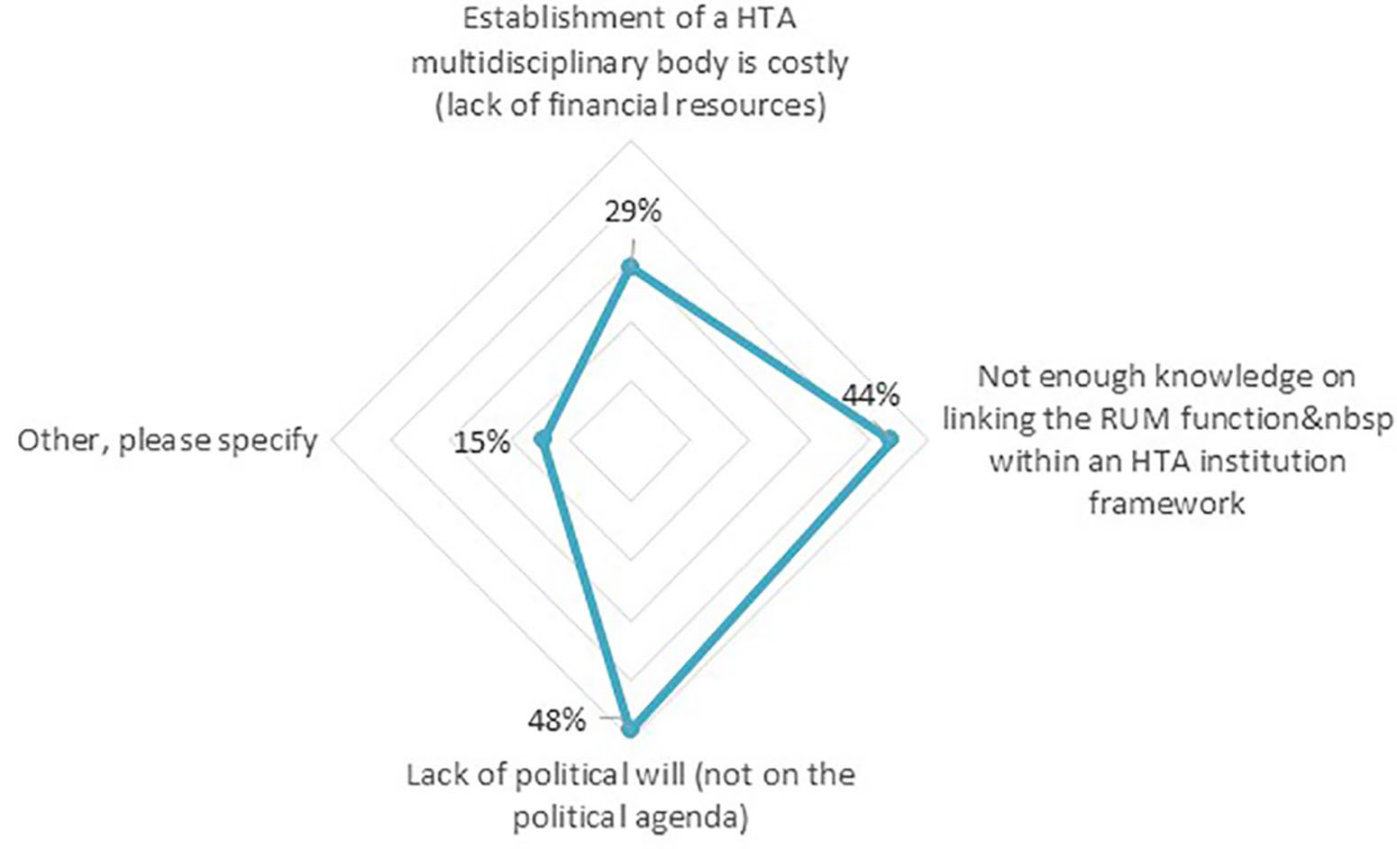
Challenges hindering the countries’ HTA entities to achieving effective RUM policy coordination (n = 62).

Our analysis shows that of the countries in which HTA plays a role in coordinating RUM initiatives, only 27% of the respondents rated their country’s HTA entity as performing above average or strong, with 31% as fair and 42% as poor or suboptimal in helping to achieve effective RUM policy co-ordination. Seventy-seven percent of the respondents subsequently believed there are certain challenges to their country’s HTA entities’ contribution to effective RUM policy coordination. The top two challenges that were mentioned (**Figure 6**) included a “lack of political will” (48%) and “Not enough knowledge in linking RUM function within an HTA institution” (44%).

To overcome these challenges, the respondents proposed solutions that fell under Education (39%) such as the inclusion of RUM-focused courses for undergraduate students within pharmacy and medical schools. Solutions pertaining to Engineering strategies (44%) were creating an HTA multidisciplinary independent body, while Economics (16%) highlighted the need for continuous funding to maintain RUM policies including potential incentives to physicians. No solutions were proposed under “Enforcement.”

### Governance of RUM Mechanisms

The majority of respondents (79%) agreed or strongly agreed that consumer education is a key intervention to enhancing RUM. However, only 16% of the respondents rated their country’s performance to consumer education as being above average or strong to promoting RUM, with 37% scoring it as fair and 47% as poor/suboptimal.

**Figure 7:**
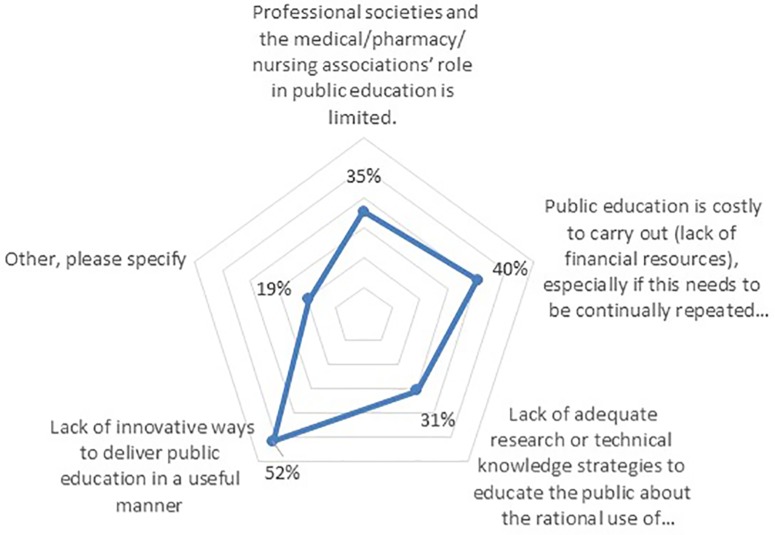
Challenges hindering achieving the key interventions to promote RUM (n = 62).

Furthermore, 74% believed there are currently challenges to providing consumer education in the context of RUM, which need to be addressed. The greatest challenge mentioned was a “lack of innovative ways to deliver public education in a useful manner” (52%), followed by “lack of financial resources” (40%), “limited role of professional societies” (35%), and “lack of technical knowledge” (31%) (**Figure 7**).

The majority of the respondents cited educational policies as a means to address the challenges that hinder effective consumer education (55%). Possible “educational” solutions that were mentioned included public campaigns *via* the local ministry of health in addition to increasing public understanding of medicines and their use. Engineering strategies such as further regulations on medicinal marketing was cited by 17% of respondents, and finally 23% cited financial resources as one strategy to support patient education around RUM.

### Role of Pan-European Groups

The top 3 organizations that respondents are aware of in the realm of RUM were WHO Europe (85%), EuNetHTA (68%), and the Piperska Group (65%) (**Table 4**), with a lower number aware of the European Association for Clinical Pharmacology and Therapeutics (EACPT—18%).

### Increasing RUM Impact

**Figure 8:**
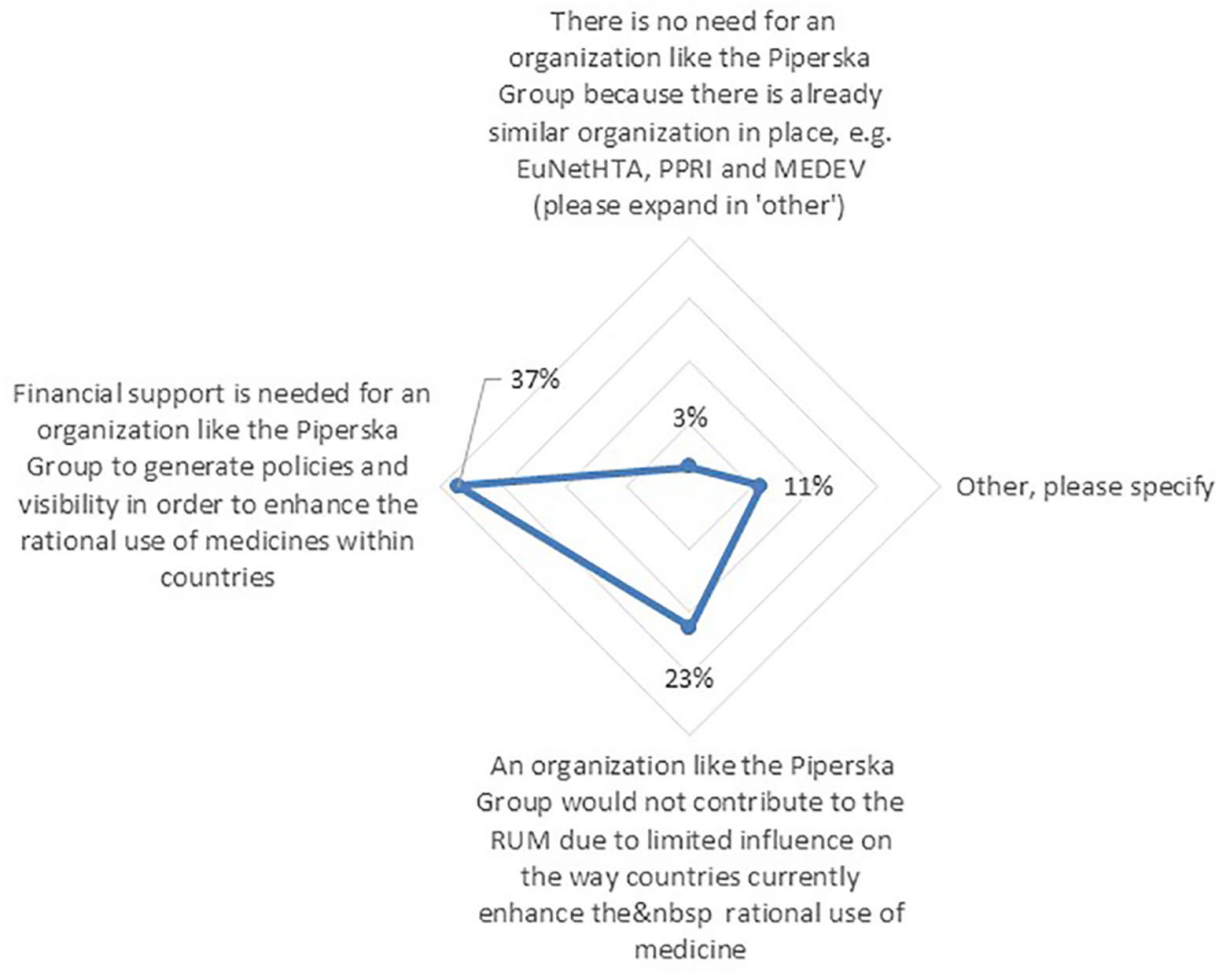
Challenges to support local entities in enhancing RUM (n = 62).

Among the respondents, 73% agreed or strongly agreed to the need for organizations that are enhancing knowledge sharing and cooperation between stakeholders across Europe with regards to RUM. However, 56% of respondents believed there are current challenges that hinder these organizations with enhancing RUM across Europe. The challenge that was mostly mentioned by respondents was “Financial support” (37%) (**Figure 8**). Finally, the majority of the solutions cited by respondents to address these challenges were again Educational policies (43%) such as communication of research results between these groups, followed by Engineering policies (28%) including strategic alliances among various pan-European groups.

## Discussion

The results of our study point to the use of clinical guidelines as the most important key intervention to enhance RUM (**Figure 1**). Whilst clinical guidelines have been instrumental in optimizing care and promoting RUM across settings (Ament et al., 2015; Colbeck et al., 2016; Ebben et al., 2018; Brouwers et al., 2019; Niaz et al., 2019; Richards et al., 2019), there are challenges such as regularly updating the guidance or sufficient government or health authority funding for their development and active dissemination in the first place. There can also be concerns with conflicts of interest among guideline developers unless adequately addressed such as the “Wise List” and guidance in Stockholm, Sweden (Gustafsson et al., 2011; Bjorkhem-Bergman et al., 2013; Shnier et al., 2016; DeJong and Steinbrook, 2018; The Lancet, 2019). Addressing the reasons behind the lack of funding for their dissemination, including how technology can assist with their wider dissemination and usability in practice to promote RUM, should be explored further. This is particularly important in situations where physician education is principally offered *via* pharmaceutical companies with the potential for bias (Spurling et al., 2010; Riaz et al., 2015; Fickweiler et al., 2017; Fadare et al., 2018; Khazzaka, 2019; Ogunleye et al., 2019), although ethical codes of practice are increasing across countries to address such concerns (Francer et al., 2014). As a result, there might be a need in some countries to develop a better understanding of mechanisms to ensure continuous updating and dissemination of clinical guidance as well as continual professional development around clinical pharmacology and RUM. In addition, ensuring in the future that clinical guidance is readily accessible in an easy-to-find manner through Apps or other mechanisms during clinic visits and other patient interactions to improve their utility. There are concerns with RUM if clinical guidelines are not readily accessible or utilized (Md Rezal et al., 2015; Mashalla et al., 2016; Matsitse et al., 2017); alternatively, where physicians are overloaded with guidelines and where they have doubts about them (Sermet et al., 2010). We do know that if physicians trust key personnel involved in the development of prescribing guidance, there is a comprehensive dissemination strategy and prescribing is regularly monitored, there is high adherence to any prescribing guidance produced. This is illustrated by high adherence rates to recommended treatment guidance in Stockholm County Council in Sweden with their “Wise List” of medicines and guidelines (Gustafsson et al., 2011; Bjorkhem-Bergman et al., 2013; Eriksen et al., 2017; Eriksen et al., 2018).

There are concerns though that DTCs recorded the lowest percentage of being key for promoting RUM (**Figure 1**). The relatively high percentage of neutral responders (24%) suggests that more awareness is needed to raise the role and function of DTCs in both ambulatory and hospital care, and its link in promoting RUM, given concerns with their implementation and effectiveness especially in LMICs (Lima-Dellamora Eda et al., 2014; Matlala et al., 2017; Fadare et al., 2018; Mashaba et al., 2019). According to the WHO, the goal of DTCs is to ensure access to the best possible cost effective and high quality of care through determining what medicines will be available at what cost and how will they be used (World Health Organization, 2003; Lima-Dellamora Eda et al., 2014). The important work of DTCs includes establishing documented rules and policies for all aspects of drug management including the selection of formulary list medicines and agreement regarding treatment protocols should be emphasized and reassessed for learnings. Other functions include continuing education for all key stakeholder groups, auditing and feedback, drug utilization reviews, and monitoring of adverse reactions and medication errors to promote RUM (Godman et al., 2009; Lima-Dellamora Eda et al., 2014; Meyer et al., 2017; Mashaba et al., 2019). Nevertheless, it still remains unclear how DTCs with this important mandate represented by these activities failed to have a critical role in promoting RUM compared to clinical guidelines. More research is needed to uncover the reasons behind this to provide future direction given the critical role that DTCs including formularies have played with enhancing the RUM (Holloway and Green, 2003; Godman et al., 2009; Thawani, 2010; Holloway et al., 2018; Sabir, 2018), and we will be exploring this further in the future.

Furthermore, there is a clear need for clarity over the linkage between HTA and RUM. It is noted that RUM is a strategy that is applied through various structures across different tiers and levels. This assumes HTA as one corner stone structure that safe-guards RUM policies. Nevertheless, the healthcare community is yet to define the nature and depth of the relationship between the two. This may be because HTA can be linked more to reimbursement and funding decisions for new medicines across countries rather than a key role in guideline development. Similar to HTA practice, RUM policies aim to optimize treatment processes and cut waste. An example of a long-lasting research program, funded by the Ministry of Health, and where HTA and RUM come together is the Rational Use of Pharmacotherapy of ZonMw in the Netherlands (ZonMw, 2015; ZonMw, 2019).

Regarding the survey results, it is imperative to reflect on how RUM interventions are carried forward by different entities and bodies within the healthcare system, and how these entities translate RUM strategies into action. Equally important is the role of HTA as a function and practice, and how HTA entities establish a harmonized relationship with the remaining structures/entities that promote RUM implementation. This consequently entails more research on establishing an effective governance system across all involved entities and stakeholders to effectively incentivize and deliver RUM interventions to improve patient care.

Finally, it is important to emphasize that educational activities remain the most important interventions to improve RUM. Compared to engineering, economics, and enforcement, education related initiatives were perceived as the most important to address challenges hindering optimal RUM. This includes raising awareness on RUM to a wider group of stakeholders including government officials, patients, practitioners, and the public. The aim would be to focus on how best to achieve better utilization of current interventions to enhance RUM, and how can these interventions be assessed, evaluated, and modified to appeal to different stakeholders as well as raise the level of understanding of the concept of RUM. These are projects for the future especially among Central and Eastern European countries.

We were aware of a number of limitations with this study. Firstly, the respondents taking part in the study were not representative of all key stakeholder groups. However, those taking part were actively involved in initiatives in their own countries to try and enhance RUM through different mechanisms. Secondly, some respondent groups had a high proportion of Piperska members. Thirdly, we did not fully query all the interventions such as “avoidance of perverse financial incentives” as we believed it’s a very complex issue that is difficult to capture through one question. Lastly, the low response rate. However, we expect the impact of a higher response rate would have been limited and would not have appreciably altered the conclusions drawn as our target sample was highly specific. Consequently, those who responded are believed to be well representative of the healthcare community involved with RUM in their country to give good guidance. As a result despite these limitations, we believe the findings provide relevant insights into current RUM initiatives and challenges within Europe with the research team seeking to maximize the survey tool to obtain results that are as accurate as possible.

## Conclusion

In conclusion, the importance of the RUM cannot be overstated given the growing pressure on healthcare resources across countries. Whilst our findings point to the importance of the key interventions surveyed, especially around clinical guideline development, the results suggest that the stakeholders are keen to develop better utilization of these interventions, investing in their appropriate application in practice. This can be achieved most prominently by increasing educational initiatives around RUM, and to call on creating mechanisms linking rational use of medicines with the HTA function already developed in most European countries. There is also a clear role for groups such as WHO Europe, especially in Central and Eastern European countries, EUNetHTA, Piperska, HTAi, EuroDURG, and patient groups such as EUPATI to enhance RUM through their various activities and initiatives. In particular, this includes encouraging countries and stakeholders to learn from each other and to collaborate in order to improve RUM building on existing Pan-European networks. We will be following this up in the future including re-evaluating progress in this area.

## Data Availability Statement

The datasets generated for this study are available on request to the corresponding author.

## Author Contributions

MG, AS, and BG developed the concept for the paper with help from WO. They subsequently coordinated the distribution of the questionnaire with the help of Jesper van den Bergh and WO. The methodology was developed by MG and AS, with principal analysis undertaken by AS and MG with all authors contributing to the development of the paper and its approval.

## Conflict of Interest

AS is employed by IQVIA.

The remaining authors declare that the research was conducted in the absence of any commercial or financial relationships that could be construed as a potential conflict of interest.
