# Supplementary material for: Mapping of Current Obstacles for Rationalizing Use of Medicines (CORUM) in Europe: Current Situation and Potential Solutions
Source: Front Pharmacol. 2020 Mar 3;11:144. doi: 10.3389/fphar.2020.00144 (PMC7063972; doi:10.3389/fphar.2020.00144)
Supplement: Supplementary file 1 [file DataSheet_1.docx]

Supplementrary Appendix 1 - CORUM Questionnaire

Introduction

The World Health Organization (WHO) estimates that more than half of all medicines are prescribed, dispensed or sold inappropriately, and that half of all patients fail to take them correctly^[[1]](#footnote-1)^. As such, a waste of scarce resources. There are also concerns with funding new medicines in view of increasing prices, and continuing pressures on resources in Europe with ageing populations, resulting in new models to address this^[[2]](#footnote-2)^ ^[[3]](#footnote-3)^. According to the WHO enhancing the appropriate use of medicines in any country involves interventions on three levels; one is on the level of healthcare authorities that includes setting strategies for monitoring their, another is on the level of healthcare service providers by following clinical guidelines, and the last being on the level of medicines use by consumers, which includes raising their awareness on the rational use of medicines.

About Piperska

The Piperska Group is a multidisciplinary network of professionals who share the common vision of enhancing the health of the public and the individual patient in a sustainable way through exchanging ideas and co-operation around the rational use of pharmacological and related therapies. The group focuses on the managed introduction of medicines, building on an acquired body of knowledge, on its members’ experience, and on evidence-based research methods. It addresses the problems that our societies are facing in maintaining and improving their health care systems. The Piperska Group pursues research and translates evidence-based results into policy recommendations. Its activities are geared towards producing recommendations ready to be employed by European and national policy makers in order to achieve their agenda of building tomorrow’s health care systems^3^, as well as running workshops. The group is comprised of researchers, practitioners from national institutions across Europe including health authority, health insurance personnel and academic, as well as patients’ representatives, all of whom are independent from industry interests.

Objectives of this survey

Country Specific Obstacles for Rationalizing the Use of Medicines in Europe (CORUM) study is aimed at qualitatively investigating the current obstacles and barriers towards enhancing the appropriate use of medicine among selected European countries. Building on previous HTA mapping studies, and other survey and study results, the objective of CORUM mapping tool is to approach relevant stakeholders in each of the target countries and record their inputs and views on the current status of the rational use of medicine in their respective countries. The study aims to highlight gaps, identify the linkage of HTA to rational use of medicines and generate cooperation between the Piperska group and relevant stakeholders to further enhance the appropriate use of medicine across Europe.

All responses will be kept confidential and only aggregated findings will be disseminated for research purposes.

**The Survey Tool**

**Contact information**

| * | Contact InformationPlease provide the details of the person integrating/completing this questionnaire: |  |
| --- | --- | --- |
|  | \| Job title \| \|  \| \| --- \| \| \| --- \| --- \| --- \| \| Organization \| \|  \| \| --- \| \| \| City/Region \| \|  \| \| --- \| \| \| Country \| \|  \| \| --- \| \| |  |
|  |  |  |

**Capacity for Rational Use of Medicine (RUM):**

| * | 1 - How strongly do you agree/disagree with the following statement being a key intervention to promote the rational use of medicines (RUM):“Use of clinical guidelines to promote RUM” | | | | |  |
| --- | --- | --- | --- | --- | --- | --- |
|  | 1 (Strongly Disagree) | 2 | 3 (Neutral) | 4 | 5 (Strongly Agree) |  |
|  |  |  |  |  |  |  |
|  |  | | | | |  |

| * | 1A - On a scale of 1-5, how would you rate your country’s performance in realizing/achieving the above statement | | | | |  |
| --- | --- | --- | --- | --- | --- | --- |
|  | 1 (Unsatisfactory) | 2 | 3 (Neutral) | 4 | 5 (Optimal) |  |
|  |  |  |  |  |  |  |
|  |  | | | | |  |

| * | 1B – Do you believe there are current limitations in achieving the above statement? |  |
| --- | --- | --- |
|  | - Yes |  |
|  | - No |  |
|  |  |  |

| * | 1C – Please state the current limitations you believe are hindering achieving this statement from the previous question (you may select multiple answers):“Use of clinical guidelines to promote RUM” |  |
| --- | --- | --- |
|  | - Lack of updated national guidelines/ prescribing guidance |  |
|  | - Lack of updated local guidelines/ prescribing guidance |  |
|  | - National guidelines are not mandated or encouraged for practical application |  |
|  | - Local guidelines are not mandated or encouraged for practical application |  |
|  | - Governments do not provide sufficient funds for the development, regular updating, and implementation of guidelines/ prescribing guidance (lack of resources) |  |
|  | - Too many national guidelines to allow for effective compliance |  |
|  | - No governance framework or legal backing for the use of guidelines |  |
|  | - Other, please specify ............................................................ |  |
|  |  |  |

| * | 1D – Please suggest a maximum of 3 potential ways to overcome the limitations you have mentioned or selected in Question 1C (if pertinent to the situation in your country/ region) |  |
| --- | --- | --- |
|  | \| 1 \| \|  \| \| --- \| \| \| --- \| --- \| --- \| \| 2 \| \|  \| \| --- \| \| \| 3 \| \|  \| \| --- \| \| |  |
|  |  |  |

| * | 2 - How strongly do you agree/disagree with the following statement being a key intervention to promote the rational use of medicines:“Development and use of national or regional formularies” | | | | |  |
| --- | --- | --- | --- | --- | --- | --- |
|  | 1 (Strongly Disagree) | 2 | 3 (Neutral) | 4 | 5 (Strongly Agree) |  |
|  |  |  |  |  |  |  |
|  |  | | | | |  |

| * | 2A - On a scale of 1-5, how would you rate your country’s performance in realizing/achieving the above statement | | | | |  |
| --- | --- | --- | --- | --- | --- | --- |
|  | 1 (Unsatisfactory) | 2 | 3 (Neutral) | 4 | 5 (Optimal) |  |
|  |  |  |  |  |  |  |
|  |  | | | | |  |

| * | 2B – Do you believe there are current limitations in achieving the above statement? |  |
| --- | --- | --- |
|  | - Yes |  |
|  | - No |  |
|  |  |  |

| * | 2C – Please state the current limitations you believe are hindering achieving this statement from the previous question (you may select multiple answers):“Development and use of national or regional formularies” |  |
| --- | --- | --- |
|  | - Lack of political will |  |
|  | - Lack of technical expertise |  |
|  | - Lack of financial resources |  |
|  | - Opposition to medicines policies by some stakeholders |  |
|  | - Lack of trust in those developing the formularies |  |
|  | - Lack of dissemination of formularies |  |
|  | - Lack of follow-up of adherence to formularies |  |
|  | - Other, please specify ............................................................ |  |
|  |  |  |

| * | 2D – Please suggest a maximum of 3 potential ways to overcome the limitations you have mentioned or selected in Question 2C (if pertinent to the situation in your country/ region) |  |
| --- | --- | --- |
|  | \| 1 \| \|  \| \| --- \| \| \| --- \| --- \| --- \| \| 2 \| \|  \| \| --- \| \| \| 3 \| \|  \| \| --- \| \| |  |
|  |  |  |

| * | 3 - How strongly do you agree/disagree with the following statement being a key intervention to promote rational use of medicines:“Establishment of drug and therapeutics committees (DTC) in districts/regions and hospitals” | | | | |  |
| --- | --- | --- | --- | --- | --- | --- |
|  | 1 (Strongly Disagree) | 2 | 3 (Neutral) | 4 | 5 (Strongly Agree) |  |
|  |  |  |  |  |  |  |
|  |  | | | | |  |

| * | 3A - On a scale of 1-5, how would you rate your country’s performance in realizing/achieving the above statement | | | | |  |
| --- | --- | --- | --- | --- | --- | --- |
|  | 1 (Unsatisfactory) | 2 | 3 (Neutral) | 4 | 5 (Optimal) |  |
|  |  |  |  |  |  |  |
|  |  | | | | |  |

| * | 3B – Do you believe there are current limitations in achieving the above statement? |  |
| --- | --- | --- |
|  | - Yes |  |
|  | - No |  |
|  |  |  |

| * | 3C – Please state the current limitations you believe are hindering achieving this statement from the previous question (you may select multiple answers):'Establishment of drug and therapeutics committees (DTC) in districts/regions and hospitals' |  |
| --- | --- | --- |
|  | - Lack of incentives and/or time for members to undertake any drug and therapeutic committee activities |  |
|  | - Lack of financial resources |  |
|  | - Lack of local expertise |  |
|  | - Drug and therapeutic committees not seen as important |  |
|  | - Lack of promoting for DTCs as organizations that can promote RUM |  |
|  | - Limited follow-up of drug and therapeutic committees recommendations |  |
|  | - Other, please specify ............................................................ |  |
|  |  |  |

| * | 3D – Please suggest a maximum of 3 potential ways to overcome the limitations you have mentioned or selected in Question 3C (if pertinent to the situation in your country/ region) |  |
| --- | --- | --- |
|  | \| 1 \| \|  \| \| --- \| \| \| --- \| --- \| --- \| \| 2 \| \|  \| \| --- \| \| \| 3 \| \|  \| \| --- \| \| |  |
|  |  |  |

| * | 4 - How strongly do you agree/disagree with the following statement being a key intervention to promote the rational use of medicines:  “Inclusion of problem-based pharmacotherapy training in undergraduate curricula at relevant institutions, and in-service continual professional development” | | | | |  |
| --- | --- | --- | --- | --- | --- | --- |
|  | 1 (Strongly Disagree) | 2 | 3 (Neutral) | 4 | 5 (Strongly Agree) |  |
|  |  |  |  |  |  |  |
|  |  | | | | |  |

| * | 4A - On a scale of 1-5, how would you rate your country’s performance in realizing/achieving the above statement | | | | |  |
| --- | --- | --- | --- | --- | --- | --- |
|  | 1 (Unsatisfactory) | 2 | 3 (Neutral) | 4 | 5 (Optimal) |  |
|  |  |  |  |  |  |  |
|  |  | | | | |  |

| * | 4B – Do you believe there are current limitations in achieving the above statement? |  |
| --- | --- | --- |
|  | - Yes |  |
|  | - No |  |
|  |  |  |

| * | 4C – Please state the current limitations you believe are hindering achieving this statement from the previous question (you may select multiple answers):“Inclusion of problem-based pharmacotherapy training in undergraduate curricula at relevant institutions, and in-service continual professional development” |  |
| --- | --- | --- |
|  | - Training programs are costly to carry out (lack of financial resources) |  |
|  | - Lack of incentives to encourage dedication to the education and training programmes by students (not a mandate for licensure) |  |
|  | - Lack of adequate research or technical knowledge to generate education and training programs on RUM. |  |
|  | - Lack of trained personnel to conduct pharmacotherapy training courses such as clinical pharmacologists and clinical pharmacists |  |
|  | - Lack of time for pharmacotherapy courses built into physician training programmes |  |
|  | - Other, please specify ............................................................ |  |
|  |  |  |

| * | 4D – Please suggest a maximum of 3 potential ways to overcome the limitations you have mentioned or selected in Question 4C (if pertinent to the situation in your country/ region) |  |
| --- | --- | --- |
|  | \| 1 \| \|  \| \| --- \| \| \| --- \| --- \| --- \| \| 2 \| \|  \| \| --- \| \| \| 3 \| \|  \| \| --- \| \| |  |
|  |  |  |

| * | 5 - How strongly do you agree/disagree with the following statement being a key intervention to promote the rational use of medicines:'Use of initiatives such as education, feedback, quality targets, and prescription restrictions (in pertinent situations) to enhance the rational use of medicines.' | | | | |  |
| --- | --- | --- | --- | --- | --- | --- |
|  | 1 (Strongly Disagree) | 2 | 3 (Neutral) | 4 | 5 (Strongly Agree) |  |
|  |  |  |  |  |  |  |
|  |  | | | | |  |

| * | 5A - On a scale of 1-5, how would you rate your country’s performance in realizing/achieving the above statement | | | | |  |
| --- | --- | --- | --- | --- | --- | --- |
|  | 1 (Unsatisfactory) | 2 | 3 (Neutral) | 4 | 5 (Optimal) |  |
|  |  |  |  |  |  |  |
|  |  | | | | |  |

| * | 5B – Do you believe there are current limitations in achieving the above statement? |  |
| --- | --- | --- |
|  | - Yes |  |
|  | - No |  |
|  |  |  |

| * | 5C – Please state the current limitations you believe are hindering achieving this statement from the previous question (you may select multiple answers):'Use of initiatives such as education, feedback, quality targets, and prescription restrictions (in pertinent situations) to enhance the rational use of medicines.' |  |
| --- | --- | --- |
|  | - Reaching a consensus (between organizations) on the appropriate initiatives to enhance RUM is difficult |  |
|  | - Lack of political will and support to instigate the initiatives or other measures to enhance RUM |  |
|  | - Lack of financial resources to fully implement programs to enhance RUM |  |
|  | - Other, please specify ............................................................ |  |
|  |  |  |

| * | 5D – Please suggest a maximum of 3 potential ways to overcome the limitations you have mentioned or selected in Question 5C (if pertinent to the situation in your country/ region) |  |
| --- | --- | --- |
|  | \| 1 \| \|  \| \| --- \| \| \| --- \| --- \| --- \| \| 2 \| \|  \| \| --- \| \| \| 3 \| \|  \| \| --- \| \| |  |
|  |  |  |

**Linkage between HTA and RUM**

| * | 6 - Does your country have a different bodies (multi-national, national, regional) that instigate and/ or coordinate RUM initiatives and policies? |  |
| --- | --- | --- |
|  | - Yes |  |
|  | - No |  |
|  |  |  |

| * | 6A - What organizations are responsible for RUM policy implementation and coordination in your country (national or regional) – please list key organisations? |  |
| --- | --- | --- |
|  | \|  \| \| --- \| |  |
|  |  |  |

| * | 6B - Please select the current type of HTA entities (unit, department, institution, organization, authority, network) in your country (national or regional) that are involved with RUM policy initiatives and/ or coordination: |  |
| --- | --- | --- |
|  | - Academia, University |  |
|  | - Funding Agency (i.e. payer) |  |
|  | - Government Agency (independent) |  |
|  | - Government National Unit (within MoH) |  |
|  | - Healthcare provider under MoH (e.g. public hospital) |  |
|  | - Healthcare provider private (e.g. private hospital) |  |
|  | - Network of HTA agencies – National/ Global (more than one country) |  |
|  | - Network of HTA Agencies – Regional (within one region) |  |
|  | - Pharmaceutical Industry (private) |  |
|  | - NGO (e.g. Fund/Foundations) |  |
|  | - Professional Society - National |  |
|  | - Professional Society – International |  |
|  | - Regulatory Authority National Government |  |
|  | - Private Insurance Agency |  |
|  | - Public Insurance Agency |  |
|  | - Other, please specify ............................................................ |  |
|  | - No HTA entity involved with RUM policy coordination |  |
|  |  |  |

| * | 6C - On a scale of 1-5, how would you rate your country’s performance in realizing/achieving effective RUM policy coordination? | | | | |  |
| --- | --- | --- | --- | --- | --- | --- |
|  | 1 (Unsatisfactory) | 2 | 3 (Neutral) | 4 | 5 (Optimal) |  |
|  |  |  |  |  |  |  |
|  |  | | | | |  |

| * | 6D – Do you believe there are current limitations in achieving the above statement? |  |
| --- | --- | --- |
|  | - Yes |  |
|  | - No |  |
|  |  |  |

| * | 6E – Please state the current limitations you believe are hindering achieving effective RUM policy coordination(you may select multiple answers): |  |
| --- | --- | --- |
|  | - Establishment of a HTA multidisciplinary body is costly (lack of financial resources) |  |
|  | - Not enough knowledge on linking the RUM function within an HTA institution framework |  |
|  | - Lack of political will (not on the political agenda) |  |
|  | - Other, please specify ............................................................ |  |
|  |  |  |

| * | 6F – Please suggest a maximum of 3 potential ways to overcome the limitations you have mentioned or selected in Question 6E (if pertinent to the situation in your country/ region) |  |
| --- | --- | --- |
|  | \| 1 \| \|  \| \| --- \| \| \| --- \| --- \| --- \| \| 2 \| \|  \| \| --- \| \| \| 3 \| \|  \| \| --- \| \| |  |
|  |  |  |

**Governance of RUM mechanisms and their practical implications**

| * | 7 - How strongly do you agree/disagree with the following statement being a key intervention to promote more rational use of medicines:'Consumer education about the use of medicines is essential to enhance their rational use, e.g. for infections and AMR” | | | | |  |
| --- | --- | --- | --- | --- | --- | --- |
|  | 1 (Strongly Disagree) | 2 | 3 (Neutral) | 4 | 5 (Strongly Agree) |  |
|  |  |  |  |  |  |  |
|  |  | | | | |  |

| * | 7A - On a scale of 1-5, how would you rate your country’s performance in realizing/achieving the above statement | | | | |  |
| --- | --- | --- | --- | --- | --- | --- |
|  | 1 (Unsatisfactory) | 2 | 3 (Neutral) | 4 | 5 (Optimal) |  |
|  |  |  |  |  |  |  |
|  |  | | | | |  |

| * | 7B – Do you believe there are current limitations in achieving the above statement? |  |
| --- | --- | --- |
|  | - Yes |  |
|  | - No |  |
|  |  |  |

| * | 7C – Please state the current limitations you believe are hindering achieving this statement from the previous question (you may select multiple answers):“Consumer education about the use of medicines is essential to enhance their rational use, e.g. for infections and AMR' |  |
| --- | --- | --- |
|  | - Professional societies and the medical/pharmacy/ nursing associations’ role in public education is limited. |  |
|  | - Public education is costly to carry out (lack of financial resources), especially if this needs to be continually repeated (e.g. antibiotics for viral infections) |  |
|  | - Lack of adequate research or technical knowledge strategies to educate the public about the rational use of medicines |  |
|  | - Lack of innovative ways to deliver public education in a useful manner |  |
|  | - Other, please specify ............................................................ |  |
|  |  |  |

| * | 7D – Please suggest a maximum of 3 potential ways to overcome the limitations you have mentioned or selected in Question 7C (if pertinent to the situation in your country/ region) |  |
| --- | --- | --- |
|  | \| 1 \| \|  \| \| --- \| \| \| --- \| --- \| --- \| \| 2 \| \|  \| \| --- \| \| \| 3 \| \|  \| \| --- \| \| |  |
|  |  |  |

**Role of PAN-Europe groups**

| * | 8 - Which of the following pan-European groups involved in advocating appropriate and efficient prescribing have you heard of? |  |
| --- | --- | --- |
|  | - Piperska group |  |
|  | - WHO Europe |  |
|  | - EuNetHTA |  |
|  | - PPRI |  |
|  | - MEDEV |  |
|  | - HTAi |  |
|  | - EACPT |  |
|  | - EuroDURG |  |
|  | - Other, please specify ............................................................ |  |
|  |  |  |

| * | 8A - Do you believe the Piperska Group plays a role in enhancing RUM? |  |
| --- | --- | --- |
|  | - Yes |  |
|  | - No |  |
|  |  |  |

| * | 8B - What role should the Piperska Group play in enhancing RUM? |  |
| --- | --- | --- |
|  | - Developing new concepts to enhance the rational use of new and existing medicines |  |
|  | - Providing governments and health authorities with expert opinions and information based on research from countries |  |
|  | - Reviewing concepts submitted by government and health authorities |  |
|  | - Other, please specify ............................................................ |  |
|  |  |  |

**Interests and Impediments to increase RUM impact**

| * | 9 - How strongly do you agree/disagree with the following statement being a key intervention to promote more rational use of medicines:'There is a need for organizations like the Piperska Group to enhance the rational use of medicines across Europe by information sharing and cooperation’’ | | | | |  |
| --- | --- | --- | --- | --- | --- | --- |
|  | 1 (Strongly Disagree) | 2 | 3 (Neutral) | 4 | 5 (Strongly Agree) |  |
|  |  |  |  |  |  |  |
|  |  | | | | |  |

| * | 9A – Do you believe there are current limitations in achieving the above statement? |  |
| --- | --- | --- |
|  | - Yes |  |
|  | - No |  |
|  |  |  |

| * | 9B – Please state the current limitations you believe are hindering achieving this statement from the previous question (you may select multiple answers):'There is a need for organizations like the Piperska Group to enhance the rational use of medicines by information sharing and cooperation’’ |  |
| --- | --- | --- |
|  | - An organization like the Piperska Group would not contribute to the RUM due to limited influence on the way countries currently enhance the rational use of medicine |  |
|  | - There is no need for an organization like the Piperska Group because there is already similar organization in place, e.g. EuNetHTA, PPRI and MEDEV (please expand in 'other') |  |
|  | - Financial support is needed for an organization like the Piperska Group to generate policies and visibility in order to enhance the rational use of medicines within countries |  |
|  | - Other, please specify ............................................................ |  |
|  |  |  |

| * | 9C – Please suggest a maximum of 3 potential ways to overcome the limitations you have mentioned or selected in Question 9B (if pertinent to the situation in your country/ region) |  |
| --- | --- | --- |
|  | \| 1 \| \|  \| \| --- \| \| \| --- \| --- \| --- \| \| 2 \| \|  \| \| --- \| \| \| 3 \| \|  \| \| --- \| \| |  |
|  |  |  |

|  | Additional comments: |  |
| --- | --- | --- |
|  | \|  \| \| --- \| |  |
|  |  |  |

1. World Health Organization, The Pursuit of Responsible Use of Medicines: Sharing and Learning from Country Experiences, 2012 [↑](#footnote-ref-1)
2. WHO. Access to new medicines in Europe: technical review of policy initiatives and opportunities for collaboration and research. 2015 [↑](#footnote-ref-2)
3. Godman B, Malmstrom RE, Diogene E et al. Are new models needed to optimize the utilization of new medicines to sustain healthcare

   systems? Expert review of clinical pharmacology. 2015;8(1):77-94 [↑](#footnote-ref-3)
